# Serum-Circulating microRNAs in Sporadic Inclusion Body Myositis

**DOI:** 10.3390/ijms241311139

**Published:** 2023-07-06

**Authors:** Matteo Lucchini, Valeria De Arcangelis, Massimo Santoro, Roberta Morosetti, Aldobrando Broccolini, Massimiliano Mirabella

**Affiliations:** 1 UOC Neurologia, Fondazione Policlinico Universitario Agostino Gemelli IRCCS, 00168 Rome, Italy; 2 Dipartimento di Neuroscienze, Sezione di Neurologia, Catholic University of Sacred Heart, 00168 Rome, Italy; 3 Energy and Sustainable Economic Development, Division of Health Protection Technologies ENEA-Italian National Agency for New Technologies, 00123 Rome, Italy

**Keywords:** inclusion body myositis, idiopathic inflammatory myopathy, miRNA, hsa-miR-192-5p, hsa-miR-372-3p

## Abstract

Background: Sporadic inclusion body myositis (s-IBM) represents a unique disease within idiopathic inflammatory myopathies with a dual myodegenerative–autoimmune physiopathology and a lack of an efficacious treatment. Circulating miRNA expression could expand our knowledge of s-IBM patho-mechanisms and provide new potential disease biomarkers. To evaluate the expression of selected pre-amplified miRNAs in the serum of s-IBM patients compared to those of a sex- and age-matched healthy control group, we enrolled 14 consecutive s-IBM patients and 8 sex- and age-matched healthy controls. By using two different normalization approaches, we found one downregulated and three upregulated miRNAs. hsa-miR-192-5p was significantly downregulated, while hsa-miR-372-3p was found to be upregulated more in the s-IBM patients compared to the level of the controls. The other two miRNAs had a very low expression levels (raw Ct data > 29). hsa-miR-192-5p and hsa-miR-372-3p were found to be significantly dysregulated in the serum of s-IBM patients. These miRNAs are involved in differentiation and regeneration processes, thus possibly reflecting pathological mechanisms in s-IBM muscles and potentially representing disease biomarkers.

## 1. Introduction

Sporadic inclusion body myositis (s-IBM) is a slowly progressive, idiopathic inflammatory myopathy (IIM) that affects males more frequently than woman. Despite it being a rare disease, with a prevalence of approximately 4–15/1,000,000, s-IBM represents the most frequent myopathy, with onset after 50 years [1,2]. Unlike other forms of IIM, s-IBM patients have little or no response to steroid therapy [3,4].

s-IBM has peculiar clinical and histological features. Clinically, s-IBM patients show the predominant involvement of finger flexors, quadriceps weakness and of swallowing muscles. Histologically, together with typical inflammatory alterations (major histocompatibility complex, MHC, a class I sarcolemmal expression with CD8 + T cells surrounding non-necrotic fibers), the main feature is represented by the presence of myodegenerative changes, as “rimmed vacuoles”, small areas of focal destruction of muscle fibres containing multi-protein aggregates [5,6]. s-IBM’s etiology remains unclear, with both degenerative and inflammatory mechanisms involved [3,4]. In the last decade, antibodies targeting cytosolic 5’-nucleotidase 1A (anti-cN1A) were found in the serum of s-IBM patients. However, this serological biomarker has low sensitivity (from 30 up to 60%) with a relatively higher specificity (compared to the other IIMs), and there is no clear-cut association with specific clinical and histological features [7,8,9].

Therefore, there is a need for more sensitive disease biomarkers that reflect the underlying pathological processes. In this context, we aim to evaluate the expression of circulating miRNAs in the serum of s-IBM patients.

miRNAs are small non-coding RNAs that are approximately 22 nucleotides in length, single-stranded and present throughout the genome in intergenic or intronic regions. miRNAs are involved in the regulation of major biological processes, including immune responses, cell differentiation, proliferation, and apoptosis; so, the pathogenic role of miRNAs has been extensively studied in malignant diseases and autoimmune disorders [10,11,12].

MiRNAs have been found in serum, plasma, and other human body fluids, indicating their high stability in the extracellular environment, suggesting that they can control cell-to-cell communication in physiological and disease situations. The extracellular circulating miRNAs have attracted attention as potential disease biomarkers. Their expression profile in body fluids, such as plasma and serum, revealed significant differences in the spectrum and concentration of miRNAs between blood and tissues, suggesting that the miRNA content in the blood may reflect specific processes associated with diseases [13].

The miRNAs expression profile of IIMs patients has been investigated in muscles, skin, and hair shaft biopsies [14,15,16,17,18,19,20,21,22,23,24]. Circulating miRNAs has been less frequently explored in IIMs, mainly in dermatomyositis and polymyositis, with only one study evaluating whole blood miRNAs expression in s-IBM patients [25].

## 2. Results

In the present study, we enrolled 14 s-IBM patients and 8 healthy volunteers. The baseline demographic features are summarized in Table 1. The healthy control group was matched for age and sex with the s-IBM group.

We analyzed 84 different miRNAs using the miScript miRNA PCR Array, including miRNAs involved both in inflammatory pathways and in muscular physiology (myomiRNAs). We chose the following criteria to evaluate miRNA expressions with the Gene globe software: the C_T_ cut-off was set to 30 cycles to avoid obtaining an overly low expression, and C. elegans miR-39 C_T_ values were used for calibration. The expression levels of the two quality controls of assay (miRTC and PPC) of each PCR array were performed. Based on the results of the assay quality check, we observed no inhibition during reverse transcription or arrays reactions.

For each method, we selected only significant changes (*p* value < 0.05) that were at least two-fold up- or downregulated as compared to those of the controls.

**Figure 1:**
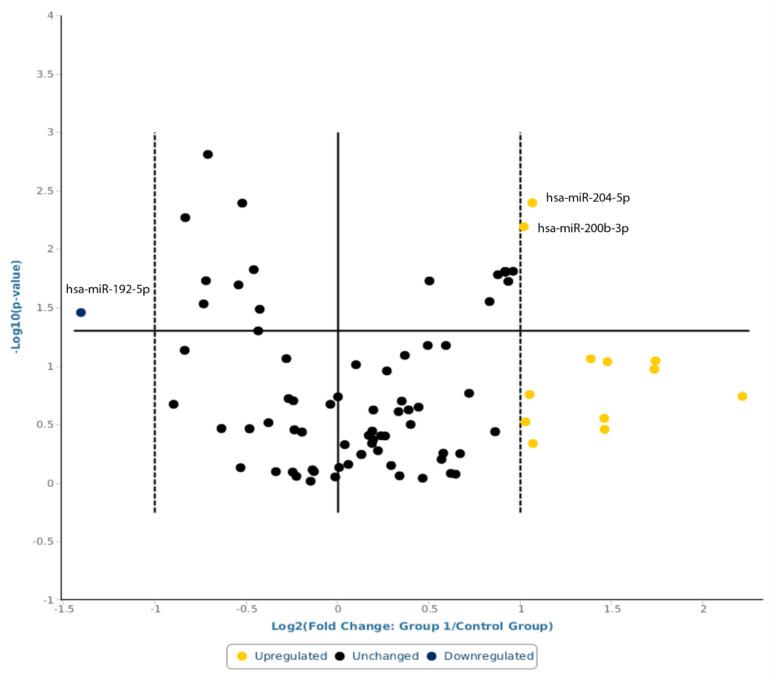
A volcano plot representation of miRNAs serum levels in sIBM patients vs. health controls using the global normalization method. Statistical significance versus fold change is shown on the y- and x-axes, respectively. Yellow circle indicates upregulation, blue circle indicates downregulation, and black circle indicates unchanged regulation.

Following the global mean normalization method, three miRs were found to be dysregulated by least two-fold in s-IBM patients compared to those of the healthy controls (Figure 1).

hsa-miR-192-5p was downregulated in s-IBM (fold change = 0.3775; 95% CI 0.14; 0.62, *p* = 0.034928), while hsa-miR-200b-3p (fold change = 2.0243; 95% CI 1.29, 2.76; 3.21; *p*= 0.006444) and hsa-miR-204-5p (fold change = 2.0905; 95% CI 1.33, 2.85; *p* = 0.004031) were found to be upregulated. However, hsa-miR-200b-3p and hsa-miR-204-5p had a very low expression level (raw Ct data were >29 in both groups).

Using the endogenous control genes method, miRNAs hsa-miR-9-5p was selected as the more stable one.

**Figure 2:**
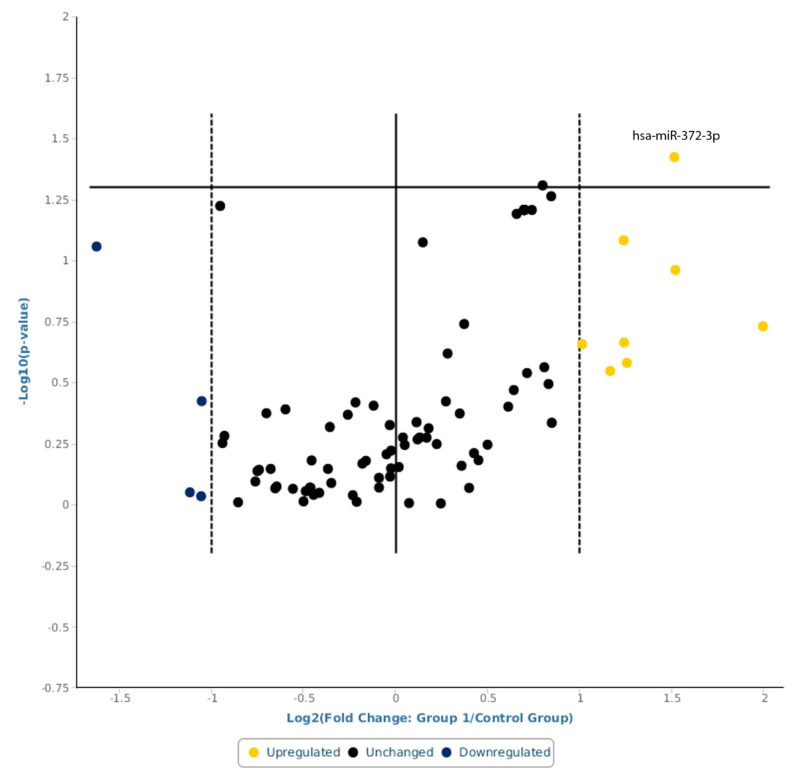
A volcano plot representation of miRNAs serum levels in sIBM patients vs. health controls using the most stably expressed miRNA normalization method (hsa-miR-9-5p). Statistical significance versus fold change is shown on the y- and x-axes, respectively. Yellow circle indicates upregulation, blue circle indicates downregulation, and black circle indicates unchanged regulation.

Via this analysis, hsa-miR-372-3p was found significantly upregulated, with a fold change higher than two (fold change = 2.8537; 95% CI 0.00001, 7.27; *p* = 0.037694) (Figure 2). hsa-miR-372-3p was found upregulated also, with global mean normalization and with a similar fold change (fold change = 3.3225; 0.00001, 6.97; *p* = 0.106529), without a statistically significance.

Ten miRNAs were not expressed in the serum samples despite the preamplification step (one or both in raw Ct data = 30). This group of miRNAs also include myomiRNAs hsa-miR-1-3p, hsa-miR-133a-3p, hsa-miR-133b and hsa-miR-206.

We performed ROC curve analysis to evaluate the diagnostic power of both hsa-miR-372-3p and hsa-miR-192-5p. The area under the curve (AUC) was 0.77 (IC 95% 0.57–0.97, *p*= 0.04) for hsa-miR-372-3p, resulting in a sensitivity of 87.5% and a specificity of 71.4%, and it was 0.74 (IC 95% 0.51–0.97, *p* = 0.08) for hsa-miR-192-5p, resulting in a sensitivity of 66.7% and a specificity of 87.5%.

### Target Prediction Analysis for microRNAs

Using the miRTargetLink 2.0 tool, we evaluated the mRNA targets for hsa-miR-192-5p and hsa-miR-372-3p. We found a strong interaction between each miRNA and several mRNA targets, 41 targets for hsa-miR-192-5p and 19 for hsa-miR-372-3p, respectively (Table 2).

From this analysis, we checked the principal pathways involved, and hsa-miR-192-5p shows a role in cytokines regulation, cell cycle checkpoints and protein metabolism, while hsa-miR-372-3p it primarily involved in cell cycle regulation. From the target mRNAs literature data, which focalized on skeletal muscle metabolism and inflammatory pathways, we found PIM1 and ZEB2 for hsa-miR-192-5p, which are involved in skeletal muscle differentiation and regeneration, and LATS2 and VEGFA for hsa-miR-372-3p, which are involved in skeletal muscle growth and aging. We also included one predicted mRNA target (NFAT5) for has-mir-192-5p using recently available literature data not included in the last update of the software [26].

## 3. Discussion

The role of miRNAs as a potential biomarker has been focused, in recent years, on their presence in human body fluids, especially in the bloodstream. The stability of miRNAs in serum and plasma makes them an easy and feasible biomarker to investigate in diagnostic applications. Their differential expression in serum or plasma compared to that in a tissue sample was of extreme importance to better understand the specific processes associated with the pathophysiological condition [16,17,18,19,20,21,22,23,24,25]. By comparing the serum expression of selected miRNAs in s-IBM patients with respect to healthy patients, we found that hsa-miR-192-5p was significantly downregulated in the serum of s-IBM patients compared to that of the control group, while hsa-miR-372-3p was significatively upregulated.

Human miR-192 is derived from a coding gene that produces two mature transcripts, miR-192 (miR-192-5p) and miR-192* (miR-192-3p) [27]. This circulating miRNA was found to be dysregulated in many diseases, including different cancers [28,29,30] and cardiocirculatory and endocrinological pathologies [31,32]. In systemic inflammation diseases, hsa-miR-192-5p is considered to be among the “circulating inflammation-related microRNAs” and found to be negatively correlated with levels of pro-inflammatory markers and cytokines, such CRP, PSP, IL-1, IL-8 and IL-6 [33].

hsa-miR-192-5p is involved in cell cycle control and cell proliferation [34]. In animal skeletal muscle development, hsa-miR-192-5p is downregulated during the myogenic differentiation of sheep satellite cells and murine C2C12 myoblasts [35]. hsa-miR-192-5p is downregulated by TGFβ-1 during the TGFβ-induced epithelial-to-mesenchymal transition through KHSRP silencing [36]. KSRP silencing in C2C12 cells induces osteoblastic differentiation and prevents myogenic differentiation [37]. The downregulation of hsa-miR-192-5p could be caused by the upregulation of TGFβ signaling that was found to be upregulated in s-IBM muscle biopsies, and its activation has been associated with inflammation, fibrosis and muscle atrophy [38,39,40,41,42].

hsa-miR-192-5p downregulation in serum could be caused by the higher rate of uptake of this miRNA by its targets in different tissues. Some of the strong targets of hsa-miR-192-5p are implicated in skeletal muscle physiology and the inflammatory pathways [43]. PIM1 regulates murine myoblast behavior because this kinase inhibition negatively influences skeletal muscle proliferation, differentiation and regeneration [44]. Another strong target, the nuclear factor of activated T cells 5 (NFAT5), was found to be downregulated by hsa-miR-192-5p in human tendon cells. This protein is a key transcription factor implicated in cellular homeostasis, immune cell survival, proliferation, migration and angiogenesis and contributes to autoimmune and inflammatory diseases. Moreover, hsa-miR-192-5p expression inhibits the NFAT5 regulation on fibrosis-related cytokines and inflammatory cytokine expression. At the same time, hsa-miR-192-5p reduces the TGF-β1-induced inflammatory response and apoptosis in tendon cells [26]. ZEB2, a predicted target for hsa-miR-192-5p, is expressed in murine skeletal muscle tissue and has an important role in the skeletal muscle differentiation of pluripotent stem cells and in muscle tissue regeneration from adult myogenic progenitors [45,46].

In this context, the serum downregulation of hsa-miR-192-5p could represent both an inflammatory and myo-regenerative biomarker of s-IBM pathology.

hsa-miR-372-3p is part of the miR-371-373 cluster that is important in stem cell biology because it was found to be expressed in human ESCs and is one of the miRNAs involved in the reprogramming of human fibroblasts to induce pluripotent stem cells [12,47,48]. Several studies underline the important role of hsa-miR-372-3p in the serum and tissues of cancer patients and cells lines, where it can be either an oncogene or an onco-suppressor [49,50,51,52,53].

hsa-miR 372-3p is implicated in the regulation of cell cycle and interacts with targets, as LATS2 or VEGF-A, involved in skeletal muscle growth, wasting and skeletal muscle fibres regeneration during aging. Since hsa-miR-372-3p was found to be upregulated in serum, we can also hypothesize another role for this miRNA. The upregulation could be explained by cells’ release in blood, indicating a possible role for hsa-miR-372-3p as a biomarker or in intercellular communication through macrovesicles delivery [54,55]. A defect in muscle reparative and regenerative mechanisms has been demonstrated in s-IBM. Moreover, mesoangioblasts isolated from s-IBM muscle biopsies have a reduced potential to differentiate into skeletal myotubes, while myoblasts show a significantly reduced proliferation rate and clonogenicity [41,56]. In this scenario, the upregulation of hsa-miR-372-3p could represent an attempt to overcome the defective muscle repair and regeneration mechanisms in s-IBM by enhancing the residual regenerative potential.

Despite a very low serum expression level, hsa-miR-200b-3p and hsa-miR-204-5p were found to be upregulated in the serum of s-IBM patients. hsa-miR-204-5p is involved in inflammation, regulating cytokine levels in type 1 diabetes and in the osteogenic differentiation of human mesenchymal stem cells.

The expression of myomiRNAs, hsa-miR-1-3p, hsa-miR-133a-3p, hsa-miR-133b and hsa-miR-206, were absent in the serum of s-IBM patients, as reported in the only other study that analyzed circulating miRNAs in whole blood [25].

Despite including the largest s-IBM cohort used so far for the evaluation of circulating miRNA, our data need further validation in a more extended cohort of patients potentially including other IIMs to evaluate whether these changes are specific for s-IBM.

In conclusion, in the serum of s-IBM patients, the downregulation of hsa-miR-192-5p and the upregulation of hsa-miR-372-3p could represent useful biomarkers related to both the inflammatory and the degenerative pathways of the disease, and their putative targets warrant more investigation to evaluate a possible pathogenic involvement in s-IBM.

## 4. Materials and Methods

### 4.1. Study Design and Participants

We consecutively enrolled patients with a clinicopathologically defined IBM based on the European Neuromuscular Centre (ENMC) 2011 research diagnostic criteria [57]. We also included a control group of healthy age- and sex-matched volunteers. Globally, we collected 22 serum samples. Exclusion criteria for both groups were having recently had an infection (within one month from sample collection), active cancer, or having been given an immunosuppressant or steroid concomitant treatment. We also excluded sIBM patients with a concurrent autoimmune or rheumatological disorders.

### 4.2. Serum Samples

Serum samples obtained from enrolled individuals were incubated at room temperature for 30 min, and then centrifuged at 1800 g for 10 min (ALC, PK121R, Aiken Corporation, CA, USA). Subsequently, serum samples were stored at −80 °C prior to use. All clinical and laboratory data were obtained at the time of serum sampling.

### 4.3. RNA Preparation, RT PCR

Total RNA was extracted from 200 μL of serum samples using an miRNeasy serum/plasma advance kit, with the addition of Spike-In Control, cel-miR-39 (3.5 uL, 1.6 × 108 copies/uL working solution) and 1 ug of bacteriophage MS2 RNA according to the manufacturer’s instructions (Qiagen^®^, Hilden, Germany). *C. elegans* miR-39 miScript primers (Qiagen^®^, Hilden, Germany) were necessary to calibrate the data sets to resolve differences in recovery that may have occurred during the purification procedure and in amplification efficiency. Calibration consists of adding the difference between the cel-miR-39 mean C_T_ of the patient and healthy control samples to every primer assay in the serum sample prior to normalization. MS2 RNA was added to improve the RNA yield during RNA extraction or isolation.

cDNA synthesis was carried out using miScript II RT Kit (Qiagen^®^, Hilden, Germany). In brief, 1.5 μL of extracted RNA was used in separate miScript RT reactions (37 °C for 60 min, and then 95 °C for 5 min, followed by 4 °C) according to the manufacturer’s instructions.

Samples were diluted 5-fold in RNase-free water prior to preamplification. All the reactions were performed using the Mastercycle Personal (Eppendorf^®^, Hamburg, Germany).

### 4.4. Preamplification and miRNA Array Analysis

Five μL of diluted cDNA was pre-amplified with miScript Preamp kit, which contained specific set of primers to target genes of miScript miRNA PCR Array Human Serum & Plasma, (Qiagen^®^, Hilden, Germany), according to the manufacturer’s instructions.

All the reactions were performed using the Mastercycle Personal (Eppendorf^®^, Hamburg, Germany). Cycling conditions were: 95 °C for 15 min, followed by 12 cycles of 30 s at 94 °C, and 3 min at 60 °C.

Amplified cDNA was diluted 20-fold in RNase-free water (Qiagen^®^, Hilden, Germany) prior to proceeding to miRNAs analysis expression with the miScript miRNA PCR array.

The difference in 84 miRsNA profiling between the healthy and patient groups was screened using the miScript miRNA PCR Array (miScript miRNA PCR Array Human Serum & Plasma, Qiagen^®^, Hilden, Germany).

The amplified cDNA was added to the miScript qPCR reaction mix containing 2× QuantiTect SYBR Green PCR master mix and the 10× miScript Universal Primer, followed by the addition of the sample to the miScript miRNA PCR array according to the manufacturer’s instructions (Qiagen^®^, Hilden, Germany).

qPCR arrays were run using StepONE PLUS Real time PCR (Thermo Fisher Scientific^®^, Waltham, MA, USA) according to the manufacturer’s recommended conditions.

The data were further analyzed using Gene globe (https://geneglobe.qiagen.com/us/analyze accessed on 16 May 2023, Qiagen^®^, Hilden, Germany).

The relative expression of mature miRNAs was calculated using the comparative CT (2^−ΔΔCt^) method, and the running of each plate was assessed via the analysis of internal plate controls. The average of the more stable miRNA among the healthy subject and patient groups was applied to the data set as the normalization factor (per plate) used to calculate the delta CT value.

We used two different approaches for normalization: global normalization [58] and the averaging of multiple endogenous control genes [59] as reported on the Qiagen Gene Globe website (https://www.qiagen.com/np/resources/faq?id=4ed1f6ff-0153-4014-b223-8073717d4ac3&lang=en accessed on 16 May 2023).

The global normalization method assumes equal RNA inputs and that the genes are expressed at a relatively stable level across the samples being compared.

The second method identifies the most stably expressed genes across all the samples. Genes that have a stability factor of less than 1.5 are considered to be stably expressed.

### 4.5. Target Prediction Analysis for microRNAs

The miRNAs hsa-miR-192-5p and hsa-miR-372-3p were analyzed using miRTargetLink 2.0 (https://ccb-compute.cs.uni-saarland.de/mirtargetlink2/unidirectional_search/ accessed on 16 May 2023) in search for their potential targets and involvement in s-IBM pathogenesis [60].

miRTargetLink is used to analyze the interaction between miRNAs and their target genes.

The validation of targets came from miRTarBase and miRATBase, and the target genes were classified as strong or weak following available literature data. Predicted targets derived from mirDIP and miRDB. In the latest database version, the authors also include annotations for pathways, miRNA sets and gene sets from miRPathDB, miEAA and GeneTrail, respectively.

### 4.6. Statistical Analyses

The *p* values were calculated based on a Student’s *t*-test of the replicate 2^(−Delta Ct) values for each gene in the healthy control group and sIBM group (https://geneglobe.qiagen.com/us/analyze, REST 2009 software v2, Gene Globe, Qiagen^®^, Hilden, Germany).

Receiver operating characteristic (ROC) curves were fitted to estimate the diagnostic performance of hsa-miR-192-5p and hsa-miR-372-3p serum expression. ROC curves were analyzed using the Statistical Package for Social Sciences, version 22.0 (IBM SPSS, Inc., Chicago, IL, USA).

## Figures and Tables

**Table 1:** Patients’ demographics.

	s-IBM Group *n* = 14	Control Group *n* = 8	*p* Value
Male sex, *n* (%)	7 (50.0)	5 (62.5)	0.571 *
Age (years)	64.6 (8.9)	63.9 (8.4)	0.859 ^#^
Disease duration (years)	1.0 (0.7)	NA	NA
Creatinkinase levels (UI/L)	721.1 (771.1)	NA	NA

All values are reported as mean (standard deviation) unless indicated otherwise. s-IBM: sporadic inclusion body myositis; UI/L: international unit/liter; * chi-squared test; ^#^ paired Student *t*-test.

**Table 2:** Predicted targets for microRNAs.

microRNA	Target	Reference/PMID
hsa-miR-192-5p	ALCAM	21119604
	BCL2	19074876, 26550150
	CDC7	19074876
	CUL5	19074876
	DLG5	19074876
	DTL	19074876
	ERCC3	19074876, 21672525
	HOXA10	19074876
	HRH1	19074876
	LMNB2	19074876
	MAD2L1	19074876
	MCM10	19074876
	MIS12	19074876
	KIF20B	19074876
	PIM1	19074876
	PRPF38A	19074876
	RACGAP1	19074876
	10-Sep	19074876
	SMARCB1	19074876
	TRAPPC2B	18835392
	WNK1	20813867
	ACVR2B	22431721
	RB1	21511813, 24012720
	ERCC4	21672525
	ATP1B1	23221637
	XIAP	25444916
	NOB1	26743688
	ITGB1	26506238
	ITGAV	26506238
	DHFR	26506238
	ESR1	27304060
	DICER1	24223844
	CAV1	24623846
	SCN5A	26209011
	NID1	25857602
	BMI1	26717043
	ITGB3	26506238
	AKT1	26351877
	H3F3A	28217257
	CD47	26506238
	SERPINE1	27216198
hsa-miR-372-3p	TRPS1	19229866
	MBNL2	19229866
	KLF13	19229866
	CDKN1A	18212054, 20190813
	LATS2	18155131, 20216554, 16564011, 22027184, 19937137
	ERBB4	19885849
	NR4A2	19885849
	VEGFA	18320040
	TGFBR2	21490602, 22020335
	RHOC	21490602
	NFIB	21608007
	CDK2	21646351, 23479742
	CCNA1	21646351
	DKK1	22020335
	BTG1	22020335
	LEFTY1	22020335
	ATAD2	24552534
	TNFAIP1	23242208
	PHLPP2	25160587

## Data Availability

The datasets generated during and/or analyzed during the current study are available from the corresponding author on reasonable request.
